# Ozone treatment promotes physicochemical properties and antioxidant capacity of fresh-cut red pitaya based on phenolic metabolism

**DOI:** 10.3389/fnut.2022.1016607

**Published:** 2022-10-06

**Authors:** Chen Li, Shan Wang, Jiayi Wang, Zhaohui Wu, Yaping Xu, Zhaoxia Wu

**Affiliations:** ^1^College of Food Science, Shenyang Agricultural University, Shenyang, China; ^2^College of Light Industry, Liaoning University, Shenyang, China; ^3^College of Life Science & Technology, Xinjiang University, Xinjiang, China; ^4^Institute of Food and Processing, Liaoning Academy of Agricultural Sciences, Shenyang, China; ^5^Chaoyang Engineering Technical School, Chaoyang, China

**Keywords:** ozone treatment, fresh-cut pitaya, quality, antioxidant activity, phenolic metabolism

## Abstract

Pitaya is an important fresh-cut product in the global fruit market. The health benefits of fresh-cut red pitaya fruit are attributed to its unique phenolic content and other antioxidants, but the fruit is highly susceptible to spoilage which causes a decline in nutritional quality. In this study, we monitored changes in quality and phenolic compounds of pitaya fruit treated with gaseous ozone during storage at 8 ± 2^°^C for 4 days. Compared with the control group, ozone treatment was an effective strategy for preserving quality by controlling the growth of microorganisms, preventing weight loss and softening, and improving the content of soluble solids and titratable acids. The results showed that ozone induced the accumulation of phenolic compounds while maintaining the quality. The content of phenolic compounds in fresh-cut pitaya was positively correlated with antioxidant activity. Ultra-performance liquid chromatography-electrospray tandem mass spectrometry was used to fingerprint the phenolic metabolites and metabolomic analysis identified 26 phenolic compounds. The majority of these were phenylpropanoids, and the key metabolic pathways were phenylpropane metabolism and flavonoid synthesis. This study illustrated the mechanism by which of ozone prolongs the shelf life of fresh-cut pitaya fruit and validated ozone as a valuable phenolic inducer and regulator of antioxidant activity, positively influencing the potential health benefits of fresh-cut products.

## Introduction

Fruits and vegetables are an important part of the human diet. They are rich in vitamins, polyphenols, minerals, and dietary fiber, and their increased consumption can reduce the risk of heart disease, colon cancer, obesity, and diabetes, and have a health promoting effect on the human body (1). A growing desire for natural products and lifestyle changes by consumers has led to an increase in the consumption rate of fresh agricultural products, thus increasing the demand for fresh-cut fruits and vegetables which are popular with consumers.

Red pitaya is fresh, healthy, nutritious, and excellent in flavor and has become an indispensable element in fresh-cut fruit plates. Recently, the fruit has become increasingly popular with consumers (2). However, compared with unprocessed fruits and vegetables, fresh-cut fruits and vegetables have a shorter shelf life and faster decline in quality because the mechanical damage during processing induces a series of physiological and metabolic changes in their tissues (3). In addition, cutting also affects the flavor and safety of fruits and vegetables because it increases ethylene production and respiration rate, water loss, color changes, tissue softening, loss of nutrients, and susceptibility to microbial contamination (4). Therefore, there is an urgent need to expand the supply of fresh-cut pitaya in the consumer market whilst maintaining nutritional quality, flavor, and safety.

Postharvest treatment plays an important role in improving the storage life of fruits and vegetables. Pulsed electric fields (5), heating (6), cold plasma technology (7), edible coating (8), and pulsed light (9) are some of the emerging technologies for postharvest treatments. Currently, attention is focused on ozone as a powerful antibacterial agent, characterized by rapid decomposition and oxidation, and is approved by the U.S. Food and Drug Administration as an antibacterial additive in direct contact with food. An appropriate concentration of ozone treatment significantly affects the storage quality of fruits and vegetables. For example, studies have reported that ozone can significantly reduce the number of microorganisms on the surface of fresh-cut cantaloupe (10), kiwi fruit (11), strawberry (12), and carrot (13), maintaining physical and chemical quality, and prolonging shelf life.

As an effective postharvest treatment for fresh-cut fruits and vegetables, ozone can activate the antioxidant defense mechanism in plant cells and metabolize reactive oxygen species (ROS), which may become an important regulator of the antioxidant potential of plant cells (14, 15). Recent studies have shown that appropriate ozone treatment can significantly increase peroxidase (POD) activity, inhibit polyphenol oxidase (PPO) activity, maintain high total phenols (TPs) and flavonoids, improve the antioxidant capacity of fruit, and maintain fruit quality (16–18). Red pitaya contains many phenolic compounds (such as anthocyanins, flavonoids, and phenolic acids), which can be used as free radical scavengers to inhibit lipid oxidation and are important antioxidants (19, 20). Therefore, it can be speculated that the antioxidant capacity of fresh-cut pitaya may be closely related to changes in phenolic compounds. Ozone has attracted extensive attention for activating the postharvest antioxidant system and inducing phenolic metabolism in fruits and vegetables. Therefore, we aimed to evaluate the effects of different ozone concentrations on prolonging the storage life, maintaining quality, and regulating the antioxidant capacity of fresh-cut pitaya based on phenolic metabolism.

## Materials and methods

### Plant material preparation

The study confirmed that 5^°^C is the critical temperature for postharvest storage of pitaya fruit. If the temperature is lower than 5^°^C, cold damage will occur (21). Red pitaya [*Hylocereus polyrhizus (Weber) Britt. & Rose*] was ordered online from its place of origin (Hainan, China). Once harvested, the fruit is transported to the laboratory by air from the cold chain (6^°^C, 85% RH) within 24 h. Pitaya was stored in a refrigerator at 8 ± 2^°^C until processing. As a result, the selected fruits had the same degree of maturity without any pests or mechanical damage. The fruit was manually peeled, cut into quarter slices (1/4 part of a 1 cm thick slice), and randomly divided into 15 cm × 10 cm × 4 cm polypropylene containers. Each container weighed approximately 150 g. The samples were stored for 4 days to simulate the actual sales process of fresh-cut fruits in China. Fresh-cut fruits are generally displayed for 1–2 days in Chinese supermarkets. After the pitaya was cut, it was immediately fumigated with ozone, and the experiment was replicated three times and stored at 8 ± 2^°^C.

### Ozone gas fumigation treatment

Ozone was prepared using a corona discharge method (22). Dried oxygen was introduced into the corona discharge pipe to obtain pure gaseous ozone. Ozone gas was released into the treatment chamber connected to the inlet and outlet, and an ozone gas concentration detector was installed in the treatment chamber. The concentration of ozone gas was controlled by adjusting the flow knob of the ozone generator, and the real-time concentration of ozone in the treatment chamber was determined using a gas concentration detector. Samples were placed in the chamber with different concentrations of ozone gas (2, 4, and 6 mg L^–1^) for 20 min. Fresh-cut pitaya fruits without ozone gas treatment were used as the control group. During storage, samples were taken every 24 h, frozen in liquid nitrogen, and stored at −80°C until use. Abbreviations for the different treatments are shown in Table 1.

**TABLE 1:** Treatmentsprepared for the experiments.

#	Treatment	Abbreviation
1	Control	CK
2	2 mg L^–1^ ozone 20 min	T1
3	4 mg L^–1^ ozone 20 min	T2
4	6 mg L^–1^ ozone 20 min	T3
5	Day 0 results for CK group	CKD0
6	Day 2 results for CK group	CKD2
7	Day 0 results for T2 group	TD0
8	Day 2 results for T2 group	TD2

### Microbial analysis

The total number of microbial colonies formed and the number of molds and yeasts were determined during storage, according to the Chinese Official Analysis Method (Chinese Official Document number: GB 4789.15-2016).

### Physicochemical properties

#### Weight loss

Weight loss is expressed as the ratio of weight loss to the initial fresh weight (%):


(1) Weight⁢loss=(initial⁢mass-observed⁢mass)/initial⁢mass×100%

#### Fruit firmness

The firmness of fresh-cut pitaya fruit was measured using a CT3 10 K texture analyzer (CT3, Brookfield, Middleboro, MA, USA) at six different positions with equal spacing from the equator. A 4 mm diameter stainless steel probe was selected, and the penetration displacement was 5 mm at a speed of 5 mm.s^–1^. The result was expressed as the maximum force (N) recorded during the penetration process.

#### Total soluble solids

Total soluble solids (TSS) was expressed as a percentage (%) using a hand-held refractometer (LB 90T, Guangzhou, China). This analysis was repeated three times; five fruits were used for each replicate.

#### Titratable acidity

Total titratable acidity (TA) was determined according to the method described by Fan et al. (2). The results were expressed as a percentage (%) of citric acid.

#### Color measurement

We measured the L*, a*, and b* values at five points on the fresh-cut pitaya using the Minolta CR-400 colorimeter (Konica Minolta Sensing, Inc., Osaka, Japan). Measurements were performed at the center of both sides of the fruit.

### Ozone treatment on antioxidants and antioxidant capacity of fresh-cut red pitaya fruit

#### Determination of total phenol content

Total phenol (TP) content was determined using the method described by Gutiérrez et al. (23). The 4 g of frozen pitaya samples were homogenized with 20 ml of methanol and 10,000 × *g* for 20 min. The supernatant of each sample was used as an extract. Results was expressed on a fresh weight (FW) basis. TP content was shown as milligrams (mg) of gallic acid per 100 g.

#### Determination of ascorbic acid content

The ascorbic acid (AsA) content was determined by titration with *2, 6*-dichlorophenolindophenol as reported by Carbone et al. (24). This analysis was repeated three times. Results were expressed on a fresh weight basis. The ascorbic acid content was expressed as milligrams (mg) per 100 g of fresh weight.

#### Determination of antioxidant capacity

Antioxidant activity was evaluated as described by Li et al. (25). The extraction method for the antioxidant activity assay was similar to that used for the total phenolic analysis (section “Determination of total phenol content”). The absorbance values were determined at 515 nm to calculate antioxidant activity using Equation 2:


(2) $$\mathrm{Antioxidant}\mathrm{activity}\left(\%\right)=\left[\left(A_{1}-A_{2}\right)/A_{1}\right]\times 100\%$$

Where A_1_ indicates the absorbance of the control and A_2_ indicates to the absorbance of the samples.

### Determination of phenolic metabolism enzyme activity

#### Enzyme solution extraction

The sample was ground into a fine paste with liquid nitrogen, then 2 g of the sample were added to 10 ml of 100 mM sodium phosphate buffer (pH 6.4, containing 0.2 g of cross-linked polyvinylpyrrolidone), centrifuged for 30 min at 4^°^C and 10,000 × *g*; further, the crude enzyme extract was the supernatant.

#### Superoxide dismutase

Superoxide dismutase (SOD) activity was determined based on the method described by Li et al. (25). One unit of SOD activity was calculated as the quantity of enzyme that caused 50% nitroblue tetrazolium reduction per second. It was expressed as U g^–1^ based on protein content.

#### Polyphenol oxidase

The activity of PPO was determined according to the method of Yingsanga et al. (26), with a slight modification. A small amount of crude enzyme extract (0.1 ml) was added to 3 ml of 100 mM catechol (prepared with sodium phosphate buffer), and then the increase in absorbance at 410 nm was measured for 3 min. Enzyme activity was expressed as ΔA 410 min^–1^ g^–1^ FW.

#### Peroxidase

Peroxidase activity was determined according to the method of Yingsanga et al. (26), with slight modifications. A small amount of crude enzyme extract (0.1 ml) was added to 2 ml of 8 mM guaiacol (prepared in sodium phosphate buffer) and incubated at 30°C for 30 min; subsequently, 1 ml of 24 mM H_2_O_2_ was added, and the increase in absorbance was measured at 470 nm for 3 min. Enzyme activity was expressed as ΔA 470 min^–1^ g^–1^ FW.

### Targeted metabolic analysis of phenolic acids and flavonoids

#### Sample preparation and extraction for metabolomic analysis

Pitaya fruits in the control group on day 0 (CKD0), day 2 (CKD2), and 4 mg L^–1^ ozone treated groups on day 0 (TD0) and day 2 (TD2) were used as experimental materials. The extraction method was as in Labadie et al. (27). The fresh-cut pitaya fruits were ground into a powder with liquid nitrogen, and the extraction and centrifugation were repeated three times at 4°C. The supernatant (200 μl) was filtered using a 0.22 μm organic phase pinhole filter, transferred to a brown glass injection vial, and stored at −80°C until liquid chromatography mass spectrometry (LC-MS) analysis.

#### Chromatography and mass spectrometry conditions

According to the method of Labadie et al. (27), the phenolic acid and flavonoid contents were determined using ultra-performance liquid chromatography-electrospray tandem mass spectrometry (UPLC-ESI-MS)/MS analysis to qualitatively and quantitatively detect the target metabolites. Therefore, various phenolic compounds were identified and quantified using reliable standards. Three technical replicates were used.

### Statistical analysis

All analyses were performed using the SPSS software package (version 20.0; SPSS, Chicago, Illinois, USA). Each treatment was repeated three times and all results are presented as the mean and the standard deviation for n replicates. The significant difference among the treatment means were compared using Tukey’s multiple range test at the 5% significance level, and the figures were created using Origin (Version 2017). *P*-values < 0.05 were considered statistically significant.

## Results

### Ozone treatment on microbial growth of fresh-cut pitaya

**FIGURE 1:**
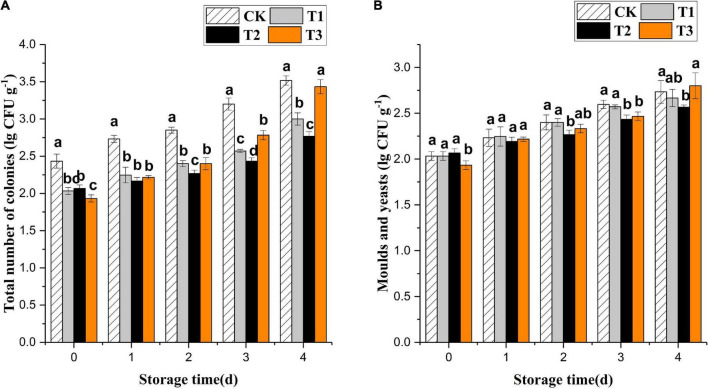
Effect of ozone treatments on total number of microbial colonies **(A)** and molds and yeasts **(B)** of fresh-cut pitaya during storage. Data were expressed as means ± standard deviations (SD) from three replications.

Ozone treatment inhibited the growth of total colonies of fresh-cut pitaya but had no significant effect on mold and yeast (Figure 1). The treatment with 4 mg L^–1^ of ozone had the best effect, which was a significantly lower growth of microbial colonies than that of the control group during storage (*P* < 0.05). At the early storage stage, the 6 mg L^–1^ treatment group showed the most apparent inhibition of total colony growth. However, the growth rate of microorganisms in the 6 mg L^–1^ treatment increased and was higher than in the 2 and 4 mg L^–1^ treatment groups on the 4th day, even though it had an edible value.

### Ozone treatment on physicochemical properties of fresh-cut pitaya

#### Ozone treatment on firmness and weight loss of fresh-cut pitaya

Firmness is directly related to fruit texture and consumer acceptance concerning the texture and storage life of fruits. The treatments with 2 and 6 mg L^–1^ of ozone resulted in fruit firmness and edible quality being maintained (Table 2). However, the firmness of untreated fruits increased in the later stages of storage.

**TABLE 2:** Effect of ozone treatment on weight loss, firmness, total soluble solids, titratable acid content, L*, a*, and b* of fresh-cut pitaya during storage.

Quality changes	Storage time (d)	Treatments			
		
		CK	T1	T2	T3
Weight loss (%)	0	0	0	0	0
	1	0.07 ± 0.02a	0.05 ± 0.01a	0.05 ± 0.01a	0.05 ± 0.01a
	2	0.13 ± 0.02a	0.08 ± 0.01b	0.09 ± 0.01b	0.09 ± 0.01b
	3	0.24 ± 0.02a	0.21 ± 0.02b	0.22 ± 0.01ab	0.23 ± 0.01ab
	4	0.31 ± 0.05a	0.26 ± 0.02b	0.26 ± 0.01ab	0.27 ± 0.01ab
Firmness (N)	0	0.36 ± 0.01a	0.38 ± 0.02a	0.36 ± 0.06a	0.34 ± 0.02a
	1	0.32 ± 0.04ab	0.29 ± 0.01b	0.39 ± 0.03a	0.31 ± 0.03b
	2	0.29 ± 0.01b	0.34 ± 0.03a	0.34 ± 0.01a	0.30 ± 0.02b
	3	0.37 ± 0.03a	0.27 ± 0.02b	0.36 ± 0.01a	0.28 ± 0.01b
	4	0.35 ± 0.02a	0.30 ± 0.02bc	0.31 ± 0.01b	0.29 ± 0.01c
TSS (%)	0	10.83 ± 0.24a	10.50 ± 0.41a	10.67 ± 0.47a	10.67 ± 0.47a
	1	10.17 ± 0.24b	10.17 ± 0.24b	10.50 ± 0.01a	10.67 ± 0.24a
	2	9.67 ± 0.24c	9.83 ± 0.24bc	10.00 ± 0.01b	10.67 ± 0.24a
	3	9.17 ± 0.24c	10.17 ± 0.24a	9.58 ± 0.12b	10.33 ± 0.24a
	4	8.33 ± 0.24d	9.67 ± 0.24b	9.17 ± 0.24c	10.17 ± 0.24a
TA (%)	0	0.41 ± 0.01a	0.41 ± 0.01a	0.43 ± 0.01a	0.41 ± 0.01a
	1	0.37 ± 0.01a	0.39 ± 0.01a	0.39 ± 0.01a	0.39 ± 0.01a
	2	0.35 ± 0.01a	0.36 ± 0.01a	0.37 ± 0.01a	0.36 ± 0.01a
	3	0.35 ± 0.01a	0.36 ± 0.01a	0.35 ± 0.01a	0.36 ± 0.01a
	4	0.34 ± 0.01a	0.35 ± 0.01a	0.35 ± 0.01a	0.35 ± 0.01a
L*	0	25.49 ± 1.54b	26.96 ± 2.15a	28.45 ± 1.83a	28.75 ± 1.08a
	1	25.25 ± 1.09b	27.34 ± 1.87a	28.21 ± 1.53a	28.50 ± 1.61a
	2	25.44 ± 1.91a	27.24 ± 2.39a	27.48 ± 1.32a	27.85 ± 1.42a
	3	25.56 ± 0.92a	26.92 ± 2.20a	28.06 ± 1.84a	28.49 ± 2.25a
	4	25.74 ± 0.98b	27.52 ± 2.85ab	27.57 ± 1.99ab	29.09 ± 0.80a
a*	0	36.71 ± 2.60a	33.75 ± 3.81a	38.91 ± 4.85a	37.66 ± 2.99a
	1	35.35 ± 2.74a	34.67 ± 3.87a	38.69 ± 4.20a	35.99 ± 3.14a
	2	35.35 ± 3.19a	34.02 ± 4.30a	36.34 ± 2.35a	34.44 ± 3.14a
	3	37.08 ± 1.21a	34.59 ± 4.42a	36.81 ± 4.28a	36.71 ± 4.20a
	4	37.07 ± 1.19a	35.25 ± 5.38a	36.77 ± 5.57a	39.13 ± 2.24a
b*	0	1.22 ± 1.25a	1.45 ± 2.02a	1.87 ± 0.79a	1.07 ± 0.43a
	1	1.14 ± 0.83a	1.48 ± 2.18a	1.70 ± 0.75a	0.87 ± 0.89a
	2	0.73 ± 1.12a	0.96 ± 1.66a	1.31 ± 1.15a	0.79 ± 0.74a
	3	1.44 ± 0.55a	1.88 ± 1.55a	2.37 ± 1.15a	1.15 ± 0.60a
	4	1.02 ± 1.30a	1.21 ± 1.65a	2.28 ± 0.91a	1.05 ± 0.46a

Data were expressed as means ± standard deviations (SD) from three replications. Means with different letters are significantly different (*P* < 0.05) at each storage period.

During storage, the weight loss of fresh-cut pitaya increased. However, compared with the control group, ozone treatment significantly inhibited weight loss during the entire storage period. The weight loss of untreated pitaya was highest (0.29%), and the inhibition effect of the 4 mg L^–1^ ozone treatment was the best (0.22%).

#### Ozone treatment on total soluble solids and titratable acidity of fresh-cut pitaya

Compared with the control group, the treatment groups delayed the decrease in soluble solids in fresh-cut pitaya and had no negative effect on titratable acid content. At the end of storage, the soluble solid content of the control group decreased by 23.8%, and that of the 4 mg L^–1^ treatment group decreased by 14.06% (Table 2).

Titratable acid represents the content of organic acids in fruits and vegetables, which directly affects the edible quality of fresh-cut pitaya fruit, such as flavor and texture. Both the control and treatment groups showed a continuous decline in TA during the storage period, while the treatment group showed a slow decline in the late storage period. The titratable acid content of fresh-cut pitaya fruit in the 4 mg L^–1^ treatment group remained the highest during storage (Table 2). Suitable ozone gas treatment can delay physiological metabolism, delaying the decline in TA content and improving the storage of fresh-cut pitaya.

#### Ozone treatment on the color of fresh-cut pitaya

The color change of fresh-cut fruits and vegetables is an important index that affects the quality of edible products and directly affects consumer acceptability. Ozone postharvest treatment and storage time had no significant effect on the color of fresh-cut pitaya, including brightness and redness (Table 2).

#### Ozone treatment on bioactive compounds and antioxidant capacity of fresh-cut pitaya

**FIGURE 2:**
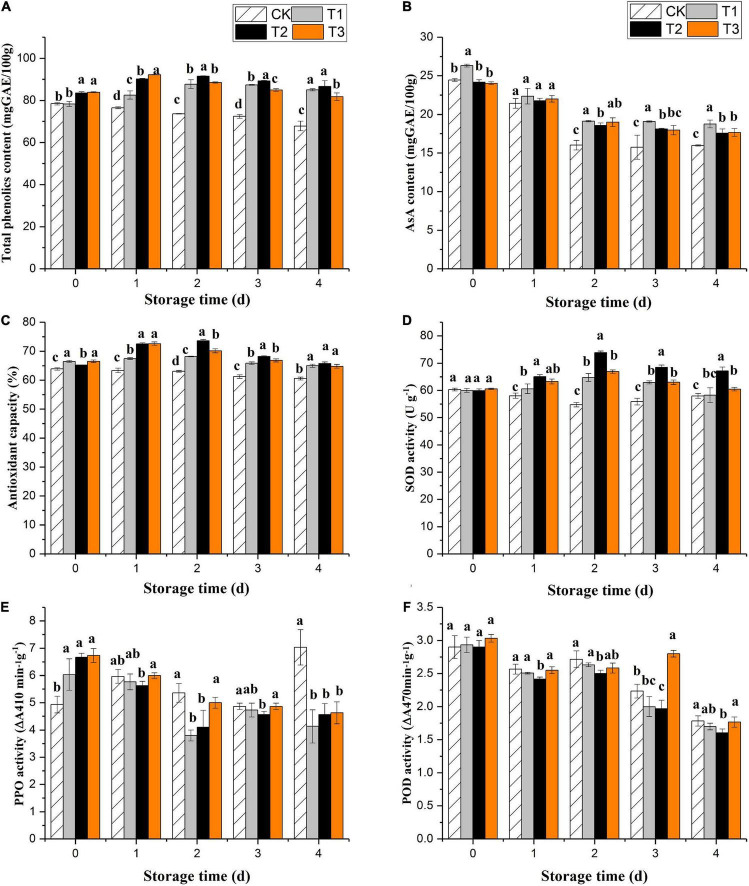
Effect of ozone treatment on total phenol **(A)**, ascorbic acid **(B)**, antioxidant capacity **(C)**, SOD activity **(D)**, PPO activity **(E)**, and POD activity **(F)** of fresh-cut pitaya during storage. Data were expressed as means ± standard deviations (SD) from three replications.

The total phenol content of the untreated fruits decreased significantly during storage. Ozone gas treatment induced phenolic accumulation in fresh-cut pitaya fruit over the first 2 days of storage, and total phenol contents reached their maximum values on the second day, increasing by 3.9, 9.5, and 9.9% for 2, 4, and 6 mg L^–1^ treatments, respectively (Figure 2A). After 2 days of storage, the fresh-cut pitaya tissues showed a decline in total phenol content, but it was still higher than the control, and the 4 mg L^–1^ treatment maintained the highest total phenol level.

All fresh-cut pitaya fruits showed a decline in the ascorbic acid content with the extension of storage time, while ozone treatment delayed the decline of ascorbic acid. On the fourth day of storage, the content of ascorbic acid in the control group decreased by 40.1%, while that in the 2, 4, and 6 mg L^–1^ ozone groups decreased by 30.0, 28.9, and 27.9%, respectively (Figure 2B). Therefore, proper ozone treatment can slow down the oxidation of ascorbic acid and maintain the nutritional quality of fresh-cut pitaya fruit.

The results showed that ozone treatment could enhance the antioxidant capacity of fresh-cut pitaya. From day 0 after ozone treatment, the antioxidant capacity of fresh-cut pitaya fruit increased, and the antioxidant activity reached its maximum value after 2 days of storage (Figure 2C).

### Ozone treatment on antioxidant enzymes and antioxidant capacity of fresh-cut pitaya

Superoxide dismutase, polyphenol oxidase, and peroxidase are key enzymes involved in the metabolism of fruits and vegetables. They are particularly important in removing reactive oxygen species (ROS), thus prolonging the shelf life of fruits and vegetables. In Figure 2D, ozone treatment induced an increase in SOD activity. Compared with the control fruit, SOD activity was still relatively high until the last day of storage. As shown in Figures 2E,F, compared with untreated fruits, ozone treatment significantly inhibited the PPO activity, but the inhibitory effect between different treatment groups was not significant. The results showed that the POD activity of the treatment and control groups decreased during storage, and there was no significant difference between the ozone treatment and control groups (*P* > 0.05).

### Correlation analysis

Table 3 shows the relationship between different attributes achieved using the Pearson correlation coefficient. Weight loss was negatively correlated with fruit firmness and TSS, and PPO activity was negatively correlated with TP. The antioxidant capacity was positively correlated with TP content and SOD activities. The results further showed that the antioxidant capacity of fresh-cut pitaya fruit was improved after ozone treatment, and phenolic compounds might play the main role.

**TABLE 3:** Pearson’s correlation coefficients between some selected quality factors of fresh-cut pitaya fruit during storage.

	Weight loss	Firmness	TSS	TA	AsA	TP	Antioxidant capacity	POD activity	SOD activity	PPO activity
Weight loss	1									
Firmness	−0.710	1								
TSS	−0.779	0.574	1							
TA	−0.797	0.718	0.654	1						
AsA	−0.821	0.764	0.789	0.848	1					
TP	−0.203	0.161	0.338	−0.058	0.176	1				
Antioxidant capacity	−0.381	0.297	0.393	0.056	0.239	0.860	1			
POD activity	−0.827	0.454	0.678	0.662	0.627	0.004	0.198	1		
SOD activity	−0.039	0.116	0.078	−0.156	−0.038	0.769	0.767	−0.090	1	
PPO activity	−0.328	0.310	0.145	0.156	0.415	−0.375	−0.215	0.326	−0.401	1

### Ozone treatment on phenolic metabolism of fresh-cut pitaya

#### Individual phenolic compounds

Twenty-six phenolic compounds were identified and quantified, including four catechin derivatives, six benzoic acid derivatives, six phenylpropanoids, three flavanols, three flavanones, two anthocyanins, and two coumarins. The initial chlorogenic acid content was 26203.59 μg kg^–1^, the highest content of phenolic compounds in fresh-cut pitaya fruit, followed by cryptochlorogenic acid, with an initial content of 1870.08 μg kg^–1^ (Table 4). The ferulic acid content and the vanillic acid content of the treatment group on day 0 was 2.46 times and 1.82 times that of the control group, respectively. Notably, on day 2, the hesperidin content of the treatment group was 30.91 times that of the control group, and the contents of rutin, narcissin, and cyanidin 3-O-rutinoside chloride in the treatment group were 10 times higher than those of the control group.

**TABLE 4:** Effects of ozone treatment on individual phenolic compounds of fresh-cut pitaya fruits during storage.

Component Name	Day 0		Day 2		Category
		
	CK	T2	CK	T2	
3,4-Dihydroxybenzaldehyde	421.26 ± 99.20ab	458.56 ± 69.23a	303.47 ± 46.60b	381.02 ± 51.12ab	Catechin derivatives
Catechin	68.14 ± 1.54b	102.47 ± 27.21a	76.91 ± 7.66ab	109.89 ± 46.47ab	Catechin derivatives
Epicatechin	32.33 ± 6.54a	42.55 ± 14.21a	18.67 ± 13.20a	74.30 ± 42.08a	Catechin derivatives
Protocatechuic acid	108.96 ± 16.34a	136.93 ± 26.85a	96.30 ± 15.83a	102.16 ± 12.25a	Catechin derivatives
Syringaldehyde	ND	16.78 ± 3.45a	10.09 ± 6.20a	ND	Benzoic acid derivatives
*trans*-Cinnamic acid	285.37 ± 180.10a	254.68 ± 20.57a	205.20 ± 100.70a	328.53 ± 222.78a	Benzoic acid derivatives
Vanillic acid	113.50 ± 16.31b	206.74 ± 59.50a	116.04 ± 38.41ab	93.39 ± 2.25c	Benzoic acid derivatives
4-Hydroxybenzoic acid	49.59 ± 5.56b	68.51 ± 9.03a	ND	30.91 ± 2.86c	Benzoic acid derivatives
Salicin	17.23 ± 2.37a	12.57 ± 0.42b	9.07 ± 1.16d	10.88 ± 0.09c	Benzoic acid derivatives
Salicylic acid	168.68 ± 7.89a	63.34 ± 15.93c	124.88 ± 5.27b	42.88 ± 0.09cd	Benzoic acid derivatives
4-Hydroxycinnamic acid	100.66 ± 41.53b	156.66 ± 1.67a	71.19 ± 20.29b	75.54 ± 6.98b	Phenylpropanoids
Caffeic acid	87.09 ± 7.60a	91.99 ± 32.71a	71.93 ± 19.29a	75.54 ± 8.17a	Phenylpropanoids
Chlorogenic acid	26203.59 ± 1958.92ab	21189.03 ± 2949.51b	22645.40 ± 2962.79b	28589.03 ± 2039.63a	Phenylpropanoids
Cryptochlorogenic acid	1870.08 ± 104.66a	1613.89 ± 236.85a	1639.37 ± 135.87a	1721.94 ± 197.00a	Phenylpropanoids
Ferulic acid	1566.84 ± 644.49ab	3848.25 ± 1732.91a	1209.96 ± 96.01b	605.83 ± 160.64c	Phenylpropanoids
Sinapic acid	881.50 ± 438.48ab	1192.94 ± 443.81a	524.18 ± 200.74b	364.88 ± 98.49b	Phenylpropanoids
Nicotiflorin	259.11 ± 84.51b	298.80 ± 61.00b	170.64 ± 70.52b	1784.04 ± 629.73a	Flavonols
Rutin	1148.77 ± 367.18bc	1455.22 ± 341.50b	736.61 ± 237.05c	7650.01 ± 164.58a	Flavonols
Narcissin	30.67 ± 10.60bc	56.64 ± 26.10b	16.57 ± 8.04c	165.68 ± 57.68a	Flavonols
Hesperidin	9.30 ± 1.06b	3.85 ± 0.32c	4.87 ± 0.83bc	150.59 ± 9.74a	Flavanones
Naringenin	2.47 ± 0.32a	2.47 ± 0.19a	2.12 ± 0.26a	2.12 ± 0.62a	Flavanones
Naringin	13.35 ± 5.17a	8.61 ± 5.05a	1.89 ± 1.20b	12.17 ± 1.95a	Flavanones
Cyanidin 3-O-rutinoside chloride	222.41 ± 61.80b	325.14 ± 79.69b	171.71 ± 76.17b	1812.35 ± 724.66a	Anthocyanins
Cyanin chloride	42.07 ± 1.80b	41.59 ± 1.40b	26.30 ± 2.82c	60.61 ± 0.90a	Anthocyanins
Aesculin	9.59 ± 0.99a	9.62 ± 1.28a	7.56 ± 1.52a	8.60 ± 1.13a	Coumarins
4-Methylumbelliferone	ND	25.30 ± 4.39	ND	ND	Coumarins

The results are expressed as μg/kg. CK represents the control group and T represents the treatment group. Data were expressed as mean ± standard deviations (SD) from three replications. Means with different letters within the same row are significantly different (*P* < 0.05) at each storage period.

#### Metabolomic analysis

**FIGURE 3:**
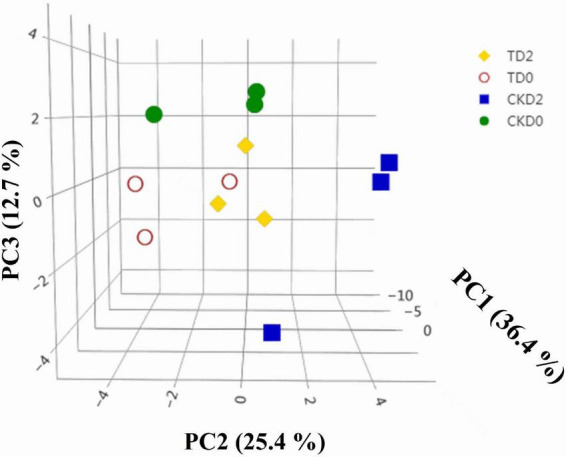
Overall sample PCA 3D score plot.

**FIGURE 4:**
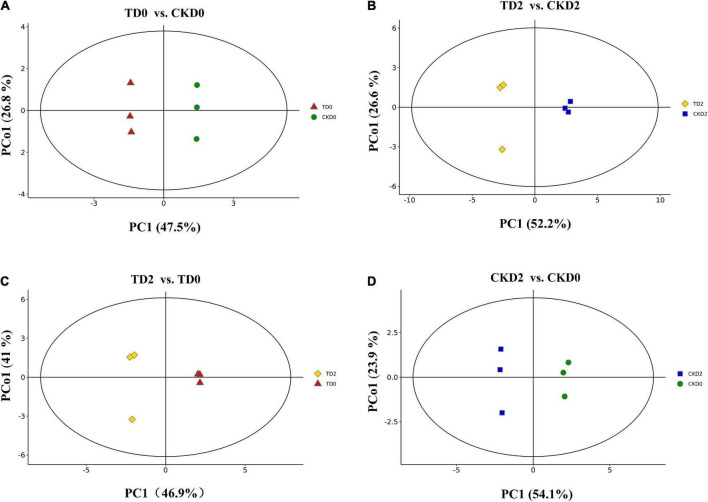
OPLS–DA model plots for the comparison groups, including TD0 vs. CKD0, TD2 vs. CKD2, TD2 vs. TD0, and CKD2 vs. CKD0 **(A–D)**.

In the present study, three principal components PC1, PC2, and PC3 were extracted (36.4, 25.4, and 12.7%, respectively) (Figure 3). In the present study, the orthogonal partial least squares discriminant analysis (OPLS–DA) model was used to perform a pairwise comparison between the samples to assess further differences; the *R*^2^ (goodness-of-fit) and *Q*^2^ (goodness-of-prediction) values of all the comparison groups were close to 1 (Figure 4). The results indicate that the data were reproducible and reliable.

**FIGURE 5:**
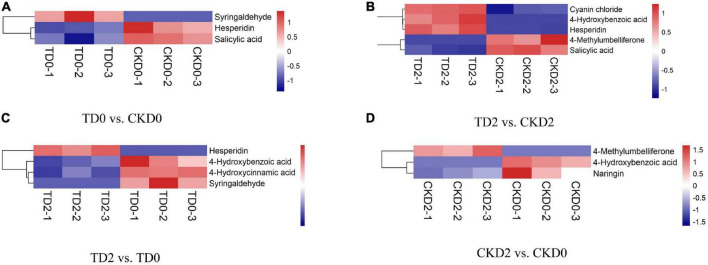
Hierarchical clustering analysis of the differential metabolites, including TD0 vs. CKD0 **(A)**, TD2 vs. CKD2 **(B)**, TD2 vs. TD0 **(C)**, and CKD2 vs. CKD0 **(D)**. Red indicates significant upregulation of metabolites, and blue indicates significant downregulation of metabolites.

In order to more visually display the relationship between samples and the expression differences of metabolites among different samples, we carried out Hierarchical Clustering on the expression levels of all significantly different metabolites and visually analyzed the expression levels of different metabolites. The results are shown in Figure 5. There were three significantly different metabolites between TD0 and CKD0 (two downregulated and one upregulated), five between TD2 and CKD2 (three downregulated and two upregulated), and four between TD2 and TD0 (3 downregulated, 1 upregulated), and 3 between CKD2 and CKD0 (2 downregulated, 1 upregulated). These significantly different metabolites were mainly derivatives of benzoic acid, flavanones, coumarins, anthocyanins, and phenylpropanoids.

**FIGURE 6:**
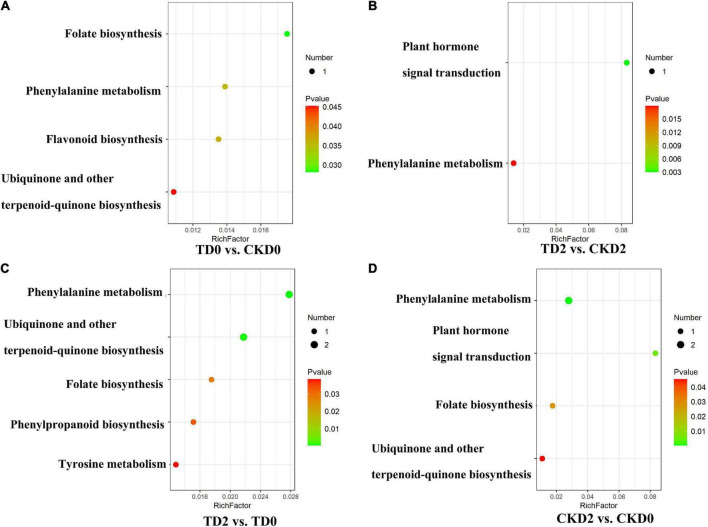
Encyclopaedia of Genes and Genomes (KEGG) enrichment plots of differential metabolites, **(A–D)** among comparison groups, including TD0 vs. CKD0, TD2 vs. CKD2, TD2 vs. TD0 and CKD2 vs. CKD0. The horizontal coordinate indicates the enrichment factor corresponding to each pathway; the vertical coordinate is the pathway name and the color of the dot is the *p*-value. The red intensity of the dot increases as the significance of the enrichment increases. The size of these dots represents the number of differential metabolites enriched.

The differential metabolites of each comparison group were annotated using the Kyoto Encyclopaedia of Genes and Genomes (KEGG) database. The above-mentioned annotated results were classified and enriched based on the pathway types in KEGG, and the enrichment results of each comparison group are shown in Figure 6. Based on the enrichment results, we observed that the differential metabolites of the comparison groups were mainly distributed in pathways including the biosynthesis of phenylalanine metabolism, ubiquinone and other terpenoid-quinone biosynthesis, folate biosynthesis, tyrosine metabolism, isoquinoline alkaloid biosynthesis, plant hormone signal transduction, and flavonoid biosynthesis.

## Discussion

Ozone is widely used as a sanitizer for storing and preserving fruits and vegetables, mainly for gaseous or aqueous ozone treatment of food products. The advantage of gaseous ozone is that it can be used as a direct antimicrobial additive to food products after diffusion in the studied system (28). Undoubtedly, fresh-cut fruits are more suitable for gaseous ozone treatment and require lower ozone concentrations compared to whole fruits. For example, room-temperature storage of raspberries at 8–10 ppm ozone oxidation for 30 min every 12 h for 3 days effectively reduced the growth of aerobic mesophilic bacteria and fungi (29). Blueberry fruit can be stored at 4°C for 28 days with ozone oxidation at 15 ppm for 30 min every 12 h, while for fresh-cut apples, it is recommended that disinfecting with 1.4 mg L^–1^ for 5 min can extend the shelf life to 10 days (18). Thus, ozone treatment varies according to the type of fruit, actual shelf life, and storage time in marketing. In addition, the ozone treatment dose was found to be a key factor affecting quality. Alwi and Ali (30) found that exposure of bell peppers to low concentrations of ozone (1 and 3 ppm) did not affect fruit respiration, color, titratable acidity, and firmness. However, exposure to ozone at 7 and 9 ppm reduced fruit quality due to excessive oxidative stress. In this study, 2, 4, and 6 mg L^–1^ ozone treatments effectively prolonged the shelf life of fresh-cut pitaya fruit compared with the control group. Therefore, it is necessary to choose an optimum ozone treatment concentration to extend the shelf life of fruits.

Ozone treatment activated protective mechanisms against antioxidant stress in fresh-cut pitaya. The levels of phenolic compounds and total antioxidant capacity were higher in fresh-cut pitaya after ozone treatment than in non-ozone-treated fruit. Zhang et al. (15) demonstrated that ozone could activate the antioxidant defense mechanism of plant cells to metabolize ROS, which may be an important regulator of superoxide anion scavenging by plant cells. In this study, we observed that ozone induced SOD enzyme activity and inhibited PPO and POD activities. Similarly, enzyme activities related to the antioxidant system, especially SOD, were induced by ozone in orange and fresh-cut green pepper (31, 32). Thus, recently, ozone has been receiving extensive attention as a suitable treatment to activate antioxidant enzyme systems in postharvest fruits and vegetables.

Many studies have revealed the potential of ozone treatment for increasing antioxidants in fruits. Fresh vegetables and fruits are often rich in antioxidant substances, such as ascorbic acid, flavonoids, and polyphenols, which are affected by ozone treatment. However, it is difficult to determine which substances play a key role in improving antioxidant activity. Therefore, we used phenolic metabolomics correlation analysis and observed that antioxidant capacity is positively correlated with total phenols, which is different from the treatment effect of green peppers. Glowacz and Rees (33) reported that ascorbic acid plays a major role because the vitamin C content of peppers is the highest among all vegetables. Ali et al. (34) reached a similar conclusion when they treated papaya fruits with 1.5, 2.5, and 3.5 ppm ozone. After 10 days of storage, the antioxidant capacity of papaya was significantly improved, and the trend obtained from DPPH and FRAP analysis was similar to the total phenol content, and phenolic compounds could be inferred as the main antioxidant in papaya. Therefore, due to the individual differences in fruits and vegetables, more research is needed to evaluate the efficacy of ozone and understand its mode of action as a postharvest treatment.

**FIGURE 7:**
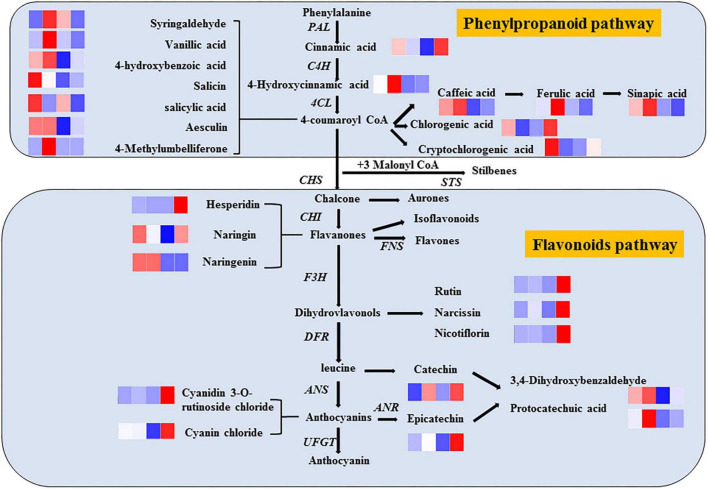
Metabolic pathways of ozone-induced phenolic accumulation in fresh-cut red pitaya fruit. The cluster heatmap showed all detected phenolic acids and flavonoid metabolites. The upregulated and downregulated metabolites were expressed by different shades of red and blue, respectively. As the abundance value increases, the color of the bar graph changes from blue to red. If the abundance value is zero, the color of the column is white.

**FIGURE 8:**
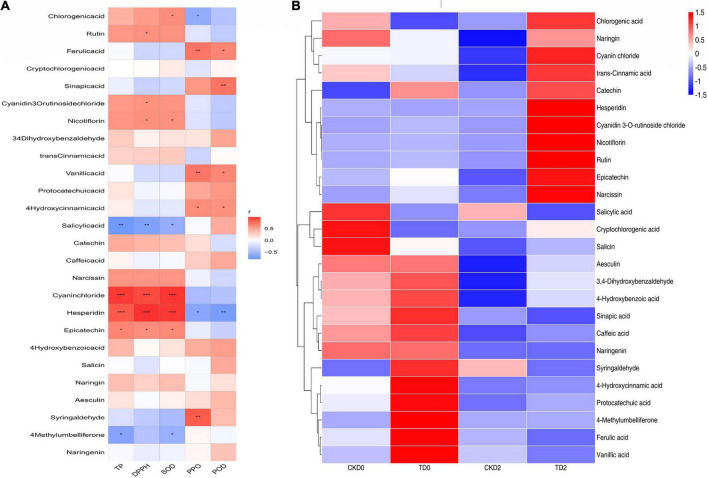
Heatmap of Spearman’s rank correlations between the individual phenolic compounds and five variables (TP, DPPH, SOD, PPO, and POD) of the fresh-cut pitaya **(A)**. The heat map of the correlation between individual phenolic compound and the storage time in fresh-cut pitaya fruit **(B)**. The significance levels are shown below: *p < 0.05; **p < 0.01; ***p < 0.001.

Phenols are widely considered the most important secondary metabolites that contribute to the antioxidant capacity of fruits (35). With the increase in phenolic compounds, the oxidation resistance of products can be greatly improved, providing us with a method to improve the efficacy of products through processing, as shown in Figure 7. Significantly, different metabolites are mainly regulated by key enzymes involved in phenanthrene metabolism and flavonoid synthesis pathways. From the expression of up- and down-regulation of significantly different metabolites, it is speculated that ozone may upregulate the expression of key enzymes in the metabolic pathway, such as anthocyanin synthase, chalcone synthase, and 4-coumaroyl-coenzyme A ligase. The heat map of the correlation between individual phenolic compound and five variables (TP, DPPH, SOD, PPO, and POD) and the storage time in fresh-cut pitaya fruit is shown in Figure 8. It can be inferred that hesperidin, 4-hydroxybenzoic acid, and cyanin chloride significantly affect the changes in phenolic substances in fresh-cut pitaya fruit, and hesperidin showed the most significant changes. Therefore, hesperidin may be a potential biomarker for ozone-induced fresh-cut pitaya fruit. Whether such a principle exists in the effect of ozone treatment on other fruits remains to be confirmed.

## Conclusion

Gaseous ozone treatment effectively maintained the sensory and nutritional properties of fresh-cut red pitaya. Postharvest ozone treatment also resulted in higher antioxidant activity, which was significantly related to changes in phenolic compound content. The regulatory effect of ozone on phenolic compounds mainly involved flavonoid biosynthesis and the biosynthesis of phenylalanine metabolism in response to ozone treatment. These findings provided new information on the effects of ozone on phenolic compounds and will help to design foods, drugs, and other products that extract health attributes from pitaya resources. However, to better understand the exact mechanism of ozone action in fresh-cut red pitaya and whether it can be used at the industrial scale, it is necessary to further study the mechanism of ozone induced phenol accumulation or oxidative stress at the molecular level.

## Data availability statement

The data analyzed in this study is subject to the following licenses/restrictions: the data that support the findings of this study are available from the corresponding author, upon reasonable request. Requests to access these datasets should be directed to ZXW, wuzxsau@163.com.

## Author contributions

CL and ZXW: conceptualization. SW: methodology and data curation. JW: software. CL, ZHW, and YX: validation and visualization. CL: formal analysis, investigation, and writing—original draft preparation and review and editing. ZXW: resources. All authors contributed to the article and approved the submitted version.
